# MINOCA: A Pathophysiological Approach of Diagnosis and Treatment—A Narrative Review

**DOI:** 10.3390/biomedicines12112457

**Published:** 2024-10-25

**Authors:** Elina Khattab, Dimitrios Karelas, Theofilos Pallas, Panagiotis Kostakis, Constantinos H. Papadopoulos, Skevos Sideris, Nikolaos Patsourakos, Nikolaos P. E. Kadoglou

**Affiliations:** 1 Medical School, University of Cyprus, 2029 Nicosia, Cyprus; 2 2nd Cardiology Department, “Korgialenio–Benakio” Red Cross Hospital, 11526 Athens, Greece; 3 Department of Cardiology, “Tzaneio” General Hospital of Piraeus, 18536 Piraeus, Greece; 4 Department of Cardiology, “Hippokration” General Hospital, 11527 Athens, Greece

**Keywords:** myocardial infarction with non-obstructive coronary arteries, thromboembolism, coronary artery spasm, spontaneous coronary artery dissection, coronary microvascular disease, diagnosis, prognosis

## Abstract

Myocardial infarction with non-obstructive coronary arteries (MINOCA) is a clinical entity characterized by the absence of significant coronary artery obstruction in epicardial arteries (<50%) on coronary angiography in the setting of acute myocardial infarction (AMI). This article aims to provide a narrative review of the pathophysiological mechanisms, diagnostic challenges, and prognosis associated with MINOCA based on pathophysiology regarding the atherosclerotic and non-atherosclerotic causes. Etiological factors, including thromboembolism, coronary artery spasm, spontaneous coronary artery dissection, coronary microvascular disease, and supply–demand mismatch, are addressed. Imaging modalities such as echocardiography, advances in coronary angiography like intravascular ultrasound (IVUS) and optical coherence tomography (OCT), cardiac magnetic resonance (CMR), and coronary computed tomography angiography (CCTA) are also analyzed. MINOCA patients have a better short-term prognosis compared to those with obstructive coronary artery disease but face significant long-term risks, underscoring the need for precise diagnosis and management strategies. Elevated inflammatory markers and specific genetic predispositions are also associated with adverse outcomes in MINOCA. This review focused on MINOCA from a pathophysiological perspective on the diverse underlying mechanisms, the challenges in achieving accurate diagnosis, the importance of a tailored therapeutic approach and the necessity for further investigation of clinical outcomes.

## 1. Introduction

Acute myocardial infarction (AMI) is one of the most extensively studied cardiovascular diseases (CVDs). In the majority of AMI cases, a significant atherosclerotic stenosis or obstruction of any epicardial coronary artery is the leading cause [1]. The performance of coronary angiography worldwide in almost all patients with AMI has led to the increasing recognition of another entity, myocardial infarction with non-obstructive coronary arteries (MINOCA). The definition of MINOCA includes all the criteria for AMI but requires non-significant stenosis in epicardial arteries (<50%) on coronary angiography [2]. Besides this, overlooking of any form of epicardial coronary artery obstruction should be excluded. Other non-atherosclerotic, non-ischemic causes of myocardial injury should be ruled out before setting MINOCA diagnosis (i.e., myocarditis, Takotsubo cardiomyopathy) [3].

**Figure 1:**
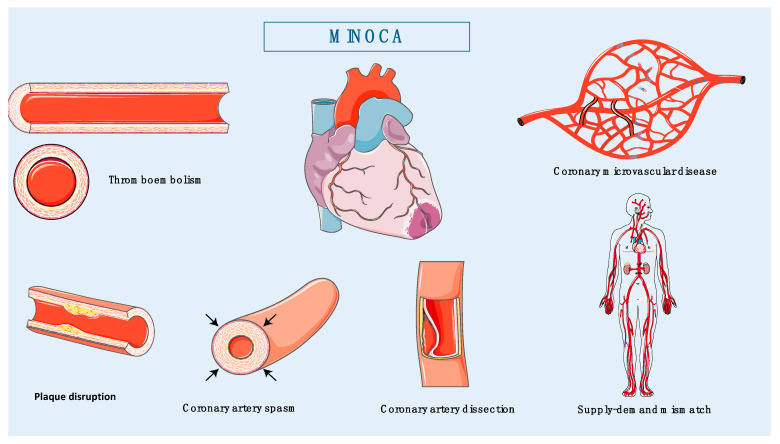
Main pathophysiological mechanisms of MINOCA.

***Causes:*** The pathophysiologic mechanisms of MINOCA vary and can be divided into cardiac and extra-cardiac, while the former is further subdivided into atherosclerotic and non-atherosclerotic origin. Common atherosclerotic mechanisms include plaque disruption or erosion, while non-atherosclerotic mechanisms include thromboembolism, epicardial coronary artery spasm (CAS), spontaneous coronary artery dissection (SCAD), coronary microvascular dysfunction (CMVD) and a mismatch between the oxygen needs and supply to the myocardium. Non-ischemic myocardial injury mimicking MINOCA typically involves situations where there is direct myocardial injury like myocarditis and cardiomyopathy Takotsubo, accompanied with troponin elevation [3]. The proposed mechanisms may be part of a systemic condition and sometimes are not fully understood (Figure 1).

***Epidemiology and prognosis:*** The prevalence of MINOCA among patients presenting with AMI typically falls within a range of 3% to 15% [4,5,6,7]. Emerging evidence highlights notable disparities in MINOCA prevalence between different etiopathogenetic mechanisms and sexes, with women being more frequently diagnosed than men [8,9]. Systematic reviews indicate an annual mortality rate of approximately 2.0% for patients with MINOCA [8]. Compared to AMI with obstructive coronary artery disease (AMI-CAD), MINOCA patients are generally younger [10], with lower all-cause mortality, but higher mortality than the CAD-free population [9,11,12,13].

***Diagnosis:*** Numerous challenges have been faced during MINOCA diagnosis due to its diverse etiologies, variable clinical presentations, and the absence of a gold-standard diagnostic modality. Coronary angiography and its advanced imaging techniques like intravascular ultrasound (IVUS) and optical coherence tomography (OCT) are the basis of the diagnostic algorithm. Usually, the heterogeneity of the MINOCA causes prompts the utilization of multiple imaging techniques. A resting echocardiography is always essential, but clinicians should consider additional modalities, like cardiac magnetic resonance (CMR) [14], functional tests of myocardial ischemia and coronary flow reserve (CFR) [15,16]. Moreover, biomarkers (e.g., for myocardial injury or inflammation) may play a role in MINOCA investigation, aiding in the comprehensive evaluation and management of patients with MINOCA [17]. In the era of genetics and artificial intelligence, precision medicine will play a pivotal role in the individualized management of MINOCA patients [18]. Overall, MINOCA is a rule-out diagnosis with a lack of standardized protocols for diagnosis and therapeutic management. The present review aims to provide a more comprehensive insight into MINOCA from the pathophysiological point of view, focusing on new indices for diagnosis, prognosis and patient-tailored treatment.

## 2. Search Strategy

We searched MEDLINE and Embase databases from January 1990 to July 2024. Our search was confined in English language publications. We used the following search terms, including Medical Subject Headings: myocardial infarction with non-obstructive coronary arteries—MINOCA; thromboembolism; coronary artery spasm; spontaneous coronary artery dissection; coronary microvascular disease; pathophysiology; echocardiography; cardiac magnetic resonance (CMR); coronary flow reserve (CFR); intravascular ultrasound (IVUS); optical coherence tomography (OCT); biomarkers; prognosis; therapy. Except for case studies, and preclinical studies (in vitro and animal), all other types of clinical studies (observational, randomized, non-randomized, prospective, retrospective) were considered eligible. The articles’ reference list was checked to identify additional relevant papers for inclusion.

## 3. Pathophysiology

The pathophysiology of MINOCA is diverse and the associated causative factors can be divided into atherosclerotic and non-atherosclerotic. For the purposes of this article, we will focus on atherosclerotic and non-atherosclerotic causes such as thromboembolism (plaque disruption and coronary embolization), epicardial CAS, SCAD, CMVD and supply–demand imbalances (Figure 1).

### 3.1. Atherosclerotic Causes

Atherosclerotic causes of MINOCA involve plaque disruption or erosion occurring without causing significant coronary artery obstruction but still leading to ischemic myocardial injury.

#### Plaque-Related Thromboembolism

Plaque-related thromboembolism plays a significant role in the pathogenesis of acute coronary syndrome (ACS), contributing to MINOCA through plaque disruption, which includes plaque rupture, erosion, and calcified nodules. These atherothrombotic events can lead to distal embolization or transient thrombotic occlusion without overt coronary artery obstruction [19,20,21,22].

Plaque Disruption: The use of high-resolution imaging modalities during coronary angiography like OCT or IVUS has revealed a high percentage of MINOCA cases linked with plaque disruption. Notably, plaque disruptions have been found in approximately one-third of MINOCA patients [23] and have been identified as a discontinuity of the fibrous cap overlying a lipid-rich core that typically occurs with superimposed thrombus formation. It is best visualized by OCT [10].

Plaque Erosion and Calcific Nodules: Plaque erosion is defined as a thrombus contiguous to the luminal surface of a plaque in the presence of an intact fibrous cap [24]. A calcific nodule defined using OCT imaging is a faint-signal region with blurry edges extending into the arterial lumen, which is a common feature in older patients with ACS but is rarely seen in MINOCA [25].

### 3.2. Non-Atherosclerotic Causes

Non-atherosclerotic causes of MINOCA involve diverse mechanisms that do not rely on the presence of atherosclerotic plaques but still result in myocardial infarction.

#### 3.2.1. Coronary Thromboembolism

Thromboembolism may be implicated as the sole intriguing factor per se in hypercoagulable states, even in the absence of atherosclerotic plaques [19,20,21]. Embolic phenomena may arise from coronary or systemic arterial thrombi in the setting of thrombophilia disorders or conditions associated with increased clotting tendency [12]. A thorough investigation for other sources of emboli should precede MINOCA diagnostic work-up in order to rule out left ventricle thrombus, vegetations or tumors. Hereditary thrombophilia like factor V Leiden mutation, protein S and C deficiencies, etc., present with varying prevalence (14–25%) among MINOCA patients, especially in young women, but they are rarely investigated [12,26,27]. The antiphospholipid syndrome, myeloproliferative diseases, thrombotic thrombocytopenic purpura (TTP) and heparin-induced thrombocytopenia (HIT) may also play a role in MINOCA presentation in the context of acquired thrombophilia disorders, but they have not been investigated thoroughly yet [28,29]. In MINOCA patients, diagnostic testing including factor V Leiden levels, prothrombin 20210A and factor VIII, as well as activities of protein C and S, antithrombin, lupus anticoagulant, and a comprehensive analysis for antiphospholipid antibodies, etc., should ideally be carried out when no other obvious reason for MINOCA is detected and thrombophilia is highly suspected (e.g., younger age, family history, without obvious cardiovascular risk factors, etc.) after the resolution of the acute phase to ensure diagnostic tests accuracy [30].

#### 3.2.2. Coronary Artery Spasm (CAS)

CAS indicates diffuse or focal severe vasoconstriction (i.e., >90%) of an epicardial coronary artery resulting in impaired myocardial blood supply [20]. Persistent CAS may lead to AMI and should also be considered in MINOCA patients. A systematic review and a prospective cohort study have demonstrated the reproducibility of episodic ischemia and chest pain using provocative tests, with rates of 27% and 46%, respectively [12,31]. CAS tends to be more common in Asian populations and its pathophysiology involves hyper-sensitivity of the vascular smooth muscle cells (VSMCs) triggered by exogenous factors like drugs or toxins (nicotine, amphetamines, cocaine) or spontaneously due to vasomotor disorders [32,33]. This hyper-reactivity is regulated by endothelium-derived signaling molecules like nitric oxide (NO) and vasoconstrictive factors released by the perivascular adipose tissue [34]. Increased Rho-kinase activity interfering with prolonged muscle contraction is also observed in patients with CAS, during active anginal episodes, and in smokers due to nicotine’s association with chronic low-grade inflammation [34]. CAS frequently occurs alongside myocardial bridging (MB), since 60% of patients with MB experienced significant vasoconstriction during provocative tests [35].

#### 3.2.3. Spontaneous Coronary Artery Dissection (SCAD)

SCAD involves the formation of a false lumen within the coronary artery wall compressing the true lumen and typically leads to ACS [36]. Most cases of SCAD arise in the absence of traditional atherosclerotic plaque formation factors and are notably more prevalent in females, but its actual incidence is unknown. In coronary angiograms, SCAD typically results in substantial (>50%) stenosis of an epicardial coronary artery, but in the minority of cases, coronary arteries might appear normal or nearly normal due to a gradual narrowing of the vessel, thereby constituting SCAD as a potential cause of MINOCA.

SCAD development is explained by two hypotheses. The “inside-out” hypothesis suggests that intimal disruption allows blood to seep into the vessel wall, forming a hematoma. On the other hand, the “outside-in” hypothesis favors hematoma formation by bleeding from the vasa vasorum without endothelial–intimal layer injury. Both hypotheses lead to a common endpoint, growing hematoma, which compresses the true lumen causing myocardial ischemia [37]. Fibromuscular dysplasia has been proposed as an alternative mechanism [38,39]. Hormonal variations and physiological changes related to pregnancy and childbirth have also been associated with alterations in the intima–media structure of the arterial wall [40]. Emotional stress, intense physical activities, and the use of sympathomimetic drugs may also play a key role in SCAD [38]. There is also a link between certain collagen vascular disorders, such as Marfan syndrome, Ehlers–Danlos syndrome, Alport syndrome, and nail–patella syndrome, as well as chronic inflammatory diseases like systemic lupus erythematosus, inflammatory bowel disease, and sarcoidosis [19]. Definitive SCAD diagnosis may require intra-vascular imaging modalities. OCT is preferable, while IVUS offers deeper penetration for visualizing the entire intramural hematoma [41].

#### 3.2.4. Coronary Microvascular Disease (CMVD)

CMVD has been long recognized as a frequent reason of ischemic symptoms and involves coronary microcirculation, with vessels smaller than 0.5 mm in diameter. These vessels represent approximately 70% of the coronary artery network and their spasm resulting in high coronary vascular resistance may lead to significant myocardial ischemia when obstructive epicardial CAD is absent [42]. CMVD is quite common among patients with MINOCA. From the pathophysiological perspective, CMVD includes either functional abnormalities with increased propensity for vasoconstriction at the microvascular level and impaired endothelium-dependent and -independent coronary vasodilator capacities, and increased coronary microvascular resistance secondary to structural factors, e.g., luminal narrowing, vascular remodeling, vascular obstruction, and extramural compression [43]. Among patients experiencing the coronary slow flow phenomenon during coronary angiogram, the elevated basal microvascular resistance plays a predominant role [44]. A small study using stress CMR on 40 female patients after MINOCA found that two-thirds exhibited inducible perfusion abnormalities, suggesting CMVD. Yet, such perfusion issues were also observed in cases of myocardial edema accompanying conditions like myocarditis. Therefore, it was unclear whether CMVD is a cause or an effect of MINOCA [45]. The main obstacle in the diagnosis of CMVD is the absence of easily performed techniques in routine clinical practice. Noninvasive tests like echocardiography CFR, stress CMR, or Positron Emission Tomography (PET) are not widely used due to time constrains, limited expertise, availability issues, high costs, or radiation risks [46]. Consequently, the contribution of CMVD to MINOCA warrants further research.

#### 3.2.5. Supply–Demand Mismatch/Type 2 Myocardial Infarction

Myocardial oxygen demand is regulated by key factors including cardiac systolic wall tension, contractility, and heart rate, while supply is mainly determined by coronary blood flow and oxygen content [47]. Type 2 AMI refers to cardiac events stemming from myocardial oxygen supply–demand mismatch. This can be seen in CAS and thrombosis or in severe systemic stresses like fast arrhythmias, anemia, severe aortic valve disease, hypotension, respiratory failure, shock (e.g., septic), heart failure or cardiomyopathy. It may also result from the adverse effects of toxins and pharmacological agents. Typically, non-obstructed, plaque-free coronary arteries are a common characteristic across all these conditions [1,48].

## 4. Diagnostic Approach Based on Pathophysiology

The AHA, ESC, and other scientific societies have outlined specific criteria and sequences for MINOCA diagnosis [20]. This is typically characterized by (1) symptoms indicative of ACS, (2) troponin elevation, and (3) non-obstructive lesions (<50%) in coronary angiography leading to a provisional diagnosis of MINOCA [15,49]. Additionally, it is a prerequisite to meticulously exclude any missed coronary obstruction and then to investigate the underlying mechanisms of myocardial injury [3,20], since MINOCA serves as a diagnostic puzzle, encompassing a diverse array of mechanisms with atherosclerotic and non-atherosclerotic origin [12,49,50]. Working diagnosis of MINOCA can indeed be challenging, and despite comprehensive evaluation, the underlying cause remains unidentified in up to 25% of patients [51,52]. Employing various advanced diagnostic modalities, such as intravascular studies (OCT, IVUS) and fractional flow reserve (FFR), as well as non-invasive techniques like CFR, cardiac computed tomography angiography (CCTA) and CMR, becomes imperative [53]. Moreover, biomarkers play a pivotal role in both diagnosis and prognosis. The emerging genetic factors and metabolomics may add depth to precision diagnosis and prognosis.

### 4.1. Imaging Modalities

#### 4.1.1. Echocardiography

Echocardiography seldom contributes to reaching the final diagnosis in MINOCA patients associated with coronary vessel dysfunction. Nonetheless, it serves as an essential initial step for assessing AMI to identify wall motion abnormalities and exclude other reasons of myocardial injury such as pericarditis and aortic dissection [54]. In MINOCA patients suspected of having CMVD, non-invasive evaluation of reduced CFR (defined as a ratio of mean blood flow < 2 between maximal hyperemia and rest) can be achieved through transthoracic Doppler [15,55]. A reduced CFR signifies the vasculature’s inability to vasodilate and increase coronary blood flow adequately to meet metabolic demands during hyperemic states. Nevertheless, its ability to discriminate CMVD from severe epicardial CAD may be sometimes limited.

#### 4.1.2. Invasive Coronary Angiography

Coronary angiography remains the initial step of MINOCA diagnostic work-up. Moreover, it is the gold standard in SCAD diagnosis, especially among young women presenting with AMI [15], while both type 2 and 3 of SCAD may necessitate better visualization with OCT or IVUS implementation for a definitive diagnosis. In case of an undetermined cause of MINOCA “functional coronary angiography”, measurement of microvascular function/coronary reactivity and intravascular imaging can be beneficial in pinpointing the underlying CMVD mechanism [49].

Provocative testing for coronary spasm should be considered during diagnostic angiography when CAS suspected [15,56]. This involves the administration of escalating doses of acetylcholine or ergonovine until it induces an epicardial coronary vasospasm [57]. The test is considered diagnostic for epicardial CAS when the following occur: (1) a reduction in the epicardial coronary diameter of ≥90% compared to the relaxed state after nitroglycerin administration, (2) reproduction of symptoms, and (3) ischemic ECG changes. However, patients with recent AMI (<6 weeks) are prone to experiencing inducible spasm during provocative testing [12] and overall, the use of functional tests in clinical practice is limited.

These provocative tests may also elicit a vasoconstrictive response at microvascular levels, implicating coronary vasomotor disorders [58]. Microvascular spasm is diagnosed when typical ischemic ECG changes occur with angina but without significant narrowing of the epicardial coronary arteries [58]. Overall, acetylcholine administration is safe with rare complications [59]. On the other hand, intracoronary administration of adenosine has limited diagnostic utility in CMVD-related MINOCA [15] because it may also indicate various reasons of myocardial injury and not exclusively MINOCA [15]. Intracoronary administration of vasodilators is crucial for diagnosing myocardial bridging, as it enhances the systolic ‘milking’ effect caused by the systolic compression of the intramural artery within the tunneled artery [58].

#### 4.1.3. Intravascular Ultrasound (IVUS) and Optical Coherence Tomography (OCT)

Intracoronary vascular imaging, including OCT and IVUS, is invaluable in assessing coronary lesions that may not be apparent on angiography, such as AMI resulting from plaque disruption, distal coronary artery embolization, and SCAD [58]. The presence of haziness in patients with a suspected culprit lesion during coronary angiography constitutes an important reason to perform IVUS or OCT, allowing both the lumen and plaque to be visualized [60]. Given the infrequent implementation of OCT or IVUS in routine practice, there is a likelihood of underdiagnosing the etiology of MINOCA cases [55]. Cost, local availability, and expertise represent potential limitations to the routine utilization of intravascular imaging [54].

IVUS is an ultrasound-based technology (~40 μm wavelength at 40 MHz) and offers a comprehensive 360-degree cross-sectional vessel image, allowing the detailed characterization of lesion morphology (e.g., ulcerations) and precise quantification of plaque burden [19,53,61]. OCT is an infrared light-based technology (1.3 μm wavelength) [61,62] and it generates images with a resolution 10 times finer (10 um) than IVUS [61], accomplishing this in a mere 2.5 s [53]. OCT, with its exceptional resolution, allows visualization of luminal and superficial coronary artery lesions and enables assessment of morphologic features at the tissue level, especially SCAD and plaque disruption [15,63]. Hybrid IVUS-OCT imaging promise improved atherosclerotic plaque characterization by minimizing imaging artifacts in both datasets, but with increasing cost.

#### 4.1.4. Fractional Flow Reserve (FFR) and Instantaneous Wave—Free Ratio (IFR)

FFR is an invasive hemodynamic functional measurement utilized to assess the significance of intermediate stenosis in epicardial coronary arteries [53]. This index is pressure-wire-based and is the gold standard invasive functional technique, since it determines whether a lesion is capable of inducing ischemia. The role and reliability of FFR in the context of MINOCA are limited and warrant further study [64]. While FFR assesses the severity of epicardial stenosis, significant microvascular disease can elevate FFR values to the same degree as an epicardial stenosis [65]. These patients may show discordance between FFR and CFR, since CFR predominantly reflects the state of the microvascular system [65]. Consequently, CFR < 2.0 and an index of microcirculatory resistance (IMR) ≥ 25 units in patients with elevated FFR denote abnormal microvascular function [65].

#### 4.1.5. Coronary Computed Tomography Angiography (CCTA)

Cardiac CT emerged as a multi-potential diagnostic tool in the assessment of MINOCA, capable of identifying various etiologies of the disease along with key prognostic factors. This imaging modality enables the assessment of extracardiac structures [53]. Most importantly, it can describe plaque characteristics like content, volume, distribution, and peri-coronary inflammation, in addition to maximal luminal stenosis [56,66]. However, it cannot identify the culprit of plaque rupture. Novel studies have utilized CCTA to characterize plaque and its inflammatory burden during the acute phase of MINOCA [53]. The peri-coronary fat inflammation, as detected by the Perivascular Fat Attenuation Index (pFAI), represents a novel imaging marker of inflammation [18,53]. Pergola et al. demonstrated that patients with MINOCA exhibited significantly elevated pFAI values compared to controls when assessed within eight days of the event, suggesting the potential utility of CCTA for identifying MINOCA cases of coronary origin [67]. Conversely, in cases of non-ischemic MINOCA, like myocarditis and takotsubo, the pFAI values remained unaltered for an extended duration. Up to now, the current evidence is insufficient to support the routine use of pAFI in MINOCA [15].

#### 4.1.6. Cardiac Magnetic Resonance (CMR)

CMR stands out as an essential test among others for MINOCA diagnosis and as a versatile imaging modality to delineate the diverse pathophysiological effects of reversible (e.g., inflammation–edema) and irreversible (e.g., necrosis–fibrosis) acute myocardial injury [68,69]. As per the ESC guidelines (2023), CMR is designated as a class I indication for all patients with MINOCA in the absence of an apparent underlying cause after invasive coronary angiography [49]. It can rule out non-MINOCA causes of troponin elevation (e.g., myocarditis, takotsubo syndrome, etc.) [49]. It constitutes a safe and non-invasive method for assessing myocardial perfusion, ventricular function, and the underlying mechanisms of myocardial injury [15]. Additionally, T2 and LGE sequences may uncover the size of myocardial infarct and abnormal diffusion patterns, enabling the differentiation between ischemic and non-ischemic injuries [19,70]. Ischemic injuries typically exhibit myocardial edema or fibrosis along vascular territories, extending either sub-endocardially or transmurally. On the other hand, myocarditis usually shows a subepicardial or mid-wall pattern [19]. CMR enables quantification of myocardial damage due to microvascular dysfunction, using first-pass perfusion (FPP) and late gadolinium enhancement (LGE) techniques [70]. A recent study demonstrated that incorporating free-breathing LGE techniques into the standard imaging protocol enabled a definitive diagnosis in 48% of MINOCA patients who initially presented with normal scans [71].

A working diagnosis of MINOCA based on intracoronary OCT followed by early CMR within 1 week of AMI presentation had 85–100% sensitivity for the identification of ischemic causes of myocardial injury. In contrast, CMR alone had a diagnostic yield of approximately 74% [54,72]. In a recent prospective, multicenter study enrolling 170 patients with MINOCA, the majority of patients where OCT was identified as the culprit lesion had abnormal CMR imaging results showing myocardial regional injury/infarction within the same coronary territory. In the future, management strategies for MINOCA of ischemic origin should combine direct intravascular imaging (e.g., OCT), and early utilization of CMR [52,73]. Finally, CMR-derived indices of myocardial perfusion like the microcirculatory perfusion index (MPI), and perfusion resistance index (MPRI) may be associated with invasive measurements and hold prognostic significance in clinical outcomes [55]. All diagnostic methods for MINOCA are summarized in Table 1.

### 4.2. Biomarkers

#### 4.2.1. Troponin

Myocardial injury is characterized by elevated cardiac troponin (cTn) levels surpassing the 99th percentile [54]. Cardiac troponin either I (cTnI) or T (cTnT) and their high-sensitivity (hs)-cTn assays stand as the preferred biomarker of myocardial injury [1]. In cases of suspected MINOCA, a thorough diagnostic assessment should rule out other clinical causes of troponin elevation, such as non-ischemic conditions (e.g., myocarditis) or noncardiac conditions (e.g., kidney impairment) [74]. cTn kinetics could serve as a distinguishing factor from AMI-CAD [17], since hs-cTn assay does not demonstrate a remarkably sharp rise in MINOCA populations compared to AMI-CAD populations [75]. Nevertheless, the troponin time curve cannot differentiate the diagnosis of MINOCA from AMI-CAD and cannot indicate the underlying cause [17].

#### 4.2.2. Inflammatory Biomarkers

Evidence suggests that MINOCA shows higher initial inflammatory activity in the acute setting, more transient effects of myocardial injury, and faster recovery compared to patients with AMI-CAD [76]. Elevated C-reactive protein (CRP) levels, the most traditional inflammatory marker, are typically observed in MINOCA patients, and they are associated with increased risk of all-cause mortality and MACE [17,77]. However, studies have failed to find any difference in CRP and hs-CRP between MINOCA and AMI-CAD patients [17]. A long list of inflammatory factors such as P-selectin glycoprotein ligand-1 (PSGL-1), interleukin 6 (IL-6), and NF-κB essential modulator (NEMO) demonstrates the exaggerated inflammatory activity in MINOCA patients [78,79,80].

MINOCA patients showed a greater increase in pro-inflammatory cytokines PlGF, oncostatin M, IL-20, and CCL-15 sVCAM-1 during the early post-infarction period and in CCL-21, sVCAM-1, oncostatin M, and PlGF after one year [81]. Notably, sVCAM-1 and CCL-21 were associated with atherosclerosis progression in MINOCA, possibly indicating complex mechanisms of microcirculatory changes. Among adipokines, visfatin is an adipocytokine abundantly produced in visceral adipose tissue and expressed in various organs [82,83]. Its potential role in endothelial dysfunction suggests its relevance to the pathogenesis of MINOCA [84]. An endless list of cytokines has been examined in MINOCA patients with potential association with MACEs, but their validation is pending. Up until now, the assessment of inflammatory burden has not been implemented in clinical practice for MINOCA management. Presumably, atherosclerotic causes may be associated with higher levels of inflammatory factors and longer time for their normalization. The prognostic value of CRP and cytokines remains to be proved.

#### 4.2.3. Natriuretic Peptides

Studies suggest that the level of N-terminal pro-B-type natriuretic peptide (NT-proBNP) was notably elevated in both MINOCA and AMI-CAD patients compared to healthy controls, with no significant difference observed between those two groups [78,80]. Their specific role in MINOCA remains elusive and its clinical application very limited.

#### 4.2.4. Metabolic Profile

Studies involving MINOCA patients demonstrated that hyperuricemia, hyperglycemia [85], and hypercholesterolemia are related to adverse outcomes and MACEs in this population [86,87]. Another study involving 1179 MINOCA patients revealed that elevated levels of Lp(a) are associated with a poorer prognosis in these individuals [88]. Elevated serum levels of Lp(a) have also been linked to microvascular injury and increased inflammatory markers, including CRP and IL-6 [89,90]. Nevertheless, it is unclear whether therapy should target those parameters. All potential biomarkers in MINOCA are summarized in Table 2.

In comparison to AMI-CAD patients, biomarker concentrations in MINOCA patients implicate a similar degree of inflammatory activity and myocardial dysfunction during the acute phase, but faster myocardial recovery. The association of biomarker levels with the underlying causes of MINOCA has not been investigated. 

### 4.3. Genetic Factors/Metabolomics

Research into the genetic foundations of cardiovascular disease pathophysiology is growing, with a particular focus on how genetic susceptibility could be a primary driver of myocardial ischemia. Studies have shown that over 50% of the risk for CAD is linked to genetic factors, primarily single-nucleotide polymorphisms (SNPs) identified through genome-wide association studies (GWASs) [91]. Another genetic study has identified molecular pathways in the vascular endothelial growth factor A (VEGF-A) and CDKN2B-AS1 genes that are linked to alterations in CFR, emphasizing the importance of these genetic variants [92]. Particularly, the CDKN2B-AS locus on chromosome 9p21, identified by GWAS, has been shown to significantly affect the proliferation and senescence of vascular smooth muscle and endothelial cells, impacting dysfunction in coronary microcirculation and interacting with inflammatory mediators [93]. Additionally, variations in the hemeoxygenase1 (HMOX1) gene, which encodes a stress-induced protective enzyme against myocardial ischemia, including SNPs with long promoter guanine-thymine repeats, have been associated with MINOCA. Genetic predisposition to microvascular dysfunction involves endothelin-1 (ET-1), a potent vasoconstrictor that modulates vascular tone and proliferation via endothelin-A (ET-A) and endothelin-B (ET-B) receptors [94]. The PHACTR1/EDN1 locus on chromosome 6p24 regulates ET-1 expression, with the rs9349379-G variant associated with elevated plasma ET-1 levels and increased risk of CAD and microvascular angina due to elevated coronary vascular resistance and impaired coronary blood flow. Further clinical trials are required [95,96].

The mechanism underlying SCAD might involve transforming growth factor-β (TGF-β) signaling, and hormonal function, which is particularly prevalent in women [17]. Mutations in genes encoding TGFBR1/2 and SMAD3 are implicated in patients with SCAD and fibromuscular dysplasia (TGF-β), Ehlers–Danlos syndrome (TGF-β1, and TGF-β2 in type IV), Loeys–Dietz syndrome (SMAD2/3, TGFBR1/2), and Marfan syndrome [97,98,99,100]. Elevated circulating TGF-β pathway proteins are observed in SCAD, but a combination of miRNAs (including miR-let-7f-5p, miR-146a-5p, miR-151a-3p, and miR-223-5p) offers better predictive values warranting further investigation [17,101]. A study found that fibrillin-1 gene (FBN1) activates integrin αvβ6/TGF-β signaling, potentially leading to endothelial cell dysfunction and SCAD [17,102]. Recent histopathology studies suggest that fibrillin-1 deficiency affects arteriole integrity, increasing vascular permeability and SCAD propensity [103]. Targeting aberrant TGF signaling, along with genetic pathways involving FBN1 and ADAMTSL4, which interacts with fibrillin-1, may provide potential therapeutic strategies [99].

Rho kinase is a downstream effector of the RhoA small GTPase, and it mediates epicardial CAS and microvascular dysfunction [104]. Activation of Rho kinase plays a crucial role in the molecular mechanisms underlying CAS. Rho kinase enhances myosin light chain (MLC) phosphorylation, leading to vascular smooth muscle cell constriction and vasospasm [105,106]. Clinical studies have demonstrated elevated Rho kinase activity in CAS patients compared to non-CAS individuals, suggesting its potential as a biomarker for CAS diagnosis and prognosis [107]. Transcriptomics studies have elucidated the complex alterations in gene expression related to myocardial ischemia/reperfusion, encompassing pathways crucial for cardiac metabolism, inflammation, and extracellular matrix remodeling. Furthermore, the identification of key miRNA targets, termed ‘protectomiRs’, holds potential for therapeutic interventions aimed at cardioprotection and cardiac regeneration in conditions such as MINOCA [108]. Transcriptomic studies have pinpointed specific miRNA clusters, such as miR15, miR17/92. The inhibition of these regulatory molecules may protect against myocardial injury [109,110]. Epigenomic and transcriptomic profiling offer quantitative insights into epigenetic changes, gene expression, and splicing variants, which are vital for studying MINOCA progression and treatment responses.

## 5. Prognosis

The cardiovascular outcomes after MINOCA have been assessed in comparison to either AMI-free individuals or patients with AMI due to significant obstruction of epicardial coronary arteries (AMI-CAD). Comparison of prognosis between MINOCA and AMI-CAD patients is challenging due to the variations in the underlying pathophysiological mechanisms and the risk profile. Unfortunately, most studies have included in their analysis patients with highly variable causes of MINOCA, meaning syndromes of atherosclerotic and non-atherosclerotic origin. Hence, the prognosis of MINOCA and its associated factors do not follow a causative stratification.

### 5.1. Comparison with Myocardial Infarction and Obstructed CAD

A systematic review has shown lower all-cause mortality at 12 months in MINOCA patients compared with AMI-CAD (4.7% vs. 6.7%) [12]. Patients with MINOCA have a significantly reduced all-cause mortality compared with those with AMI-CAD, including a 63% lower in-hospital mortality and 41% lower 12-month mortality. Although these findings may be reassuring, the 4.7% (95% CI, 2.6–6.9%) of 12-month all-cause mortality for patients with MINOCA is of concern when compared with other published prognostic studies [12]. On the other hand, the KAMIR-NIH registry demonstrated similar 2-year all-cause death comparing MINOCA and AMI-CAD (9.1% versus 8.8%) [4]. This is a very high mortality rate after AMI for patients with non-obstructive CAD and highlights the importance of recognizing and treating MINOCA based on its etiology. However, a recent retrospective analysis of patients from the ACUITY study, involving 13,800 patients with moderate-to-high-risk ACS who underwent coronary angiography within 72 h, showed that compared to NSTEMI patients, MINOCA patients had a higher risk of one-year mortality (4.7% vs. 3.6%), although associated with an increase in non-cardiac deaths (2.1% vs. 1.2%), against a higher rate of recurrent AMI and repeated revascularizations in NSTEMI patients at one year [111]. It is well known that mortality increases along with CAD severity; however, the prognosis of MINOCA patients may be equivalent to those without multi-vessel disease. Firstly, the Korean MI Registry [112] evaluated 12-month all-cause mortality in 8510 consecutive AMI patients, reporting a 3.1% annual mortality in those with MINOCA, 3.2% in those with single- or double-vessel coronary artery disease, and 6.5% in those with triple-vessel disease or a significant left main coronary artery stenosis. Secondly, Ciliberti et al. [113] hypothesized that the worse prognosis in AMI-CAD patients than their MINOCA counterparts is mainly affected by the presence of multi-vessel or left main disease.

In addition to mortality, the history of MINOCA is associated with higher cardiovascular morbidity. The GENESIS-PRAXIS study revealed that, despite the absence of obstructive CAD, MINOCA patients may have high risk characteristics and approximately 14% of MACEs occur within 1 year of follow-up [114]. More notably, the results from the SWEDEHEART registry showed that 23.9% of MINOCA patients experienced MACEs over a 4-year follow-up period [115].

### 5.2. Comparison with General Population

The prognosis of MINOCA patients should be considered somewhat guarded despite being better than those with AMI-CAD because it remains poorer than AMI-free subjects. Notably, both short- and long-term survival rates of MINOCA patients are lower than in the general population [115]. Patients with no previous AMI, but stable chest pain and normal/smooth coronaries on angiography, have a 0.2% annual all-cause mortality, whereas those with only minor luminal irregularities have a 0.3% annual all-cause mortality, which is by far lower than MINOCA patients [4].

In the largest study to date, Andersson et al. [116] compared long-term survival and causes of death in STE-ACS patients with and without obstructive CAD; the main findings were that STE-ACS patients without obstructive CAD (i) had better short-term survival but similar or worse long-term survival compared with patients with obstructive CAD, (ii) had worse short- and long-term survival compared with people in the general population, and (iii) mainly died of non-cardiovascular causes of death. Perhaps, the degree of cardiac injury during MINOCA may still increase the risk for adverse events.

### 5.3. Determinants of MINOCA Prognosis

There is a limited number of studies on MINOCA’s prognostic risk factors, and it is not clear if they differ from the relative factors in AMI-CAD patients. A previous systematic review has shown that a reduced LV ejection fraction, nonobstructive CAD, β-blockers during follow-up, and ST depression on ECG at admission are independent predictors of worse long-term prognosis of MINOCA patients [8]. The KAMIR-NIH study [2] concluded that, in MINOCA patients, old age, classical symptoms, ST interval elevation on ECG, Killip Class IV, and diabetes were independent predictors of all-cause death at the 2-year follow-up. Older age, higher creatinine concentration, low LV ejection fraction and ST elevation have also been linked to increased mortality [6,8]. Various observational studies have added a plethora of potential independent predictors of MACEs in the MINOCA population, including history of hypertension, diabetes, smoking, female gender, atrial fibrillation, elevated creatinine levels and history of any of the following: stroke, AMI, peripheral vascular disease, heart failure and chronic obstructive pulmonary disease [117,118,119].

Intriguingly, some angiographic findings may be associated with worse prognosis. For instance, non-obstructive coronary atherosclerosis appears to be associated with poorer outcomes in MINOCA compared to patients devoid of evident atherosclerotic plaques [56,120]. In parallel, individuals with coronary slow flow demonstrate a heightened incidence of MACEs than those with normal coronary flow rates. Patients with positive acetylcholine testing exhibited significantly worse long-term prognoses compared to those with negative results, suggesting that MINOCA patients with abnormal vasoreactivity face a heightened risk of future cardiovascular events [73].

Regarding the prognostic value of biomarkers, elevated hs-cTn levels constitute an independent risk factor for MACEs [121] and increased mortality (ACUITY trial) [104] in MINOCA patients. Moreover, elevated plasma levels of IL-6 have been associated with adverse cardiovascular outcomes [78,79], or atherothrombotic events in MINOCA, due to CMVD or coronary artery spasm [122].

Unfortunately, the prognosis of MINOCA stratified by the underlying mechanism is mostly unknown. Most studies have investigated the impact of MINOCA in mixed populations of various causes due to the small number of patients assigned to each cause. For instance, SCAD is a rare cause of MINOCA and it is practically impossible to draw a firm conclusion on its impact on prognosis [123]. A multicenter registry of Quesada O et al. (2023) showed even worse 5-year mortality in the group of 120 MINOCA patients presented with STEMI compared to the 8151 cohort of STEMI patients with obstructive coronary arteries (HR: 1.93, 95% CI, 1.06–3.53) [124]. The MINOCA cohort included coronary artery plaque disruption, CAS, SCAD without obstruction and coronary embolism/thrombosis. Therefore, future multicenter trials will unravel the link between MINOCA causes and prognosis.

## 6. Current Management of MINOCA

Since MINOCA has many plausible pathological mechanisms, it is still uncertain whether the classical treatment strategy for secondary prevention for type 1 AMI is suitable for MINOCA patients. On 27 March 2019, the American Heart Association (AHA) released guidelines for the diagnosis and management of MINOCA [20]; according to those recommendations, risk stratification and the most appropriate treatment scheme should be selected on the basis of etiology. However, most of the data were derived from observational studies and require further investigation. For instance, a previous, large-scale observational study in Sweden [115] found significant proportions of patients after MINOCA on statins, angiotensin-converting enzyme inhibitors/angiotensin receptor blockers (ACEI/ARB), β-blockers, and dual antiplatelet therapy (DAPT): 84.5%, 64.1%, 83.4%, and 66.4%, respectively. Notably, in the same study 23.9% of patients experienced a MACE during an average follow-up of 4.1 years.

### 6.1. ACEI/ARB and Statins

The risk of experiencing a MACE was 18% lower (HR, 0.82; 95% CI, 0.73–0.93) in patients with ACEI/ARB in comparison with no ACEI/ARB; 23% lower (HR, 0.77; 95% CI, 0.68–0.87) in patients with statins versus statins-free patients; and 14% lower after β-blocker usage (HR, 0.86; 95% CI, 0.74–1.01) than in patients without β-blockers. Hence, this study [115] showed that after MINOCA, both statins and ACEI/ARB had a long-term beneficial effect on the outcome, while β-blockers showed a positive trend. In agreement, the KAMIR-NIH study [4] showed that long-term treatment of MINOCA patients with ACEI and statins was associated with reduced mortality after a 2-year follow-up. Similar results from the EMMACE-2 study showed the association of ACEI with reduced 6-month mortality (HR 0.31, 95% CI 0.03–0.78, *p* < 0.004) after MINOCA [123]. On the other hand, statins did not reduce MACEs in MINOCA patients [125].

### 6.2. Anti-Platelet Therapy

The SWEDEHEART registry documented a non-significant benefit after 1 year of DAPT therapy (HR, 0.90; 95% CI, 0.74–1.08). Other studies have shown neutral or even harmful effects of antiplatelet treatment to MINOCA patients, implicating its suspension in daily practice [126]. Ishii et al. [127] found that long-term use of aspirin after discharge due to MINOCA could not reduce adverse CV events. Other studies have shown that intensive clopidogrel therapy tends to be associated with an increased risk of CV mortality, AMI, and stroke in MINOCA patients [126]. In particular, a subgroup analysis of a randomized trial of antiplatelet strategies (with MINOCA being 6.7% of enrolled patients) compared high- vs. low-dose clopidogrel, and high- vs. low-dose aspirin. This study demonstrated that a higher dose of clopidogrel was associated with poorer outcomes than a standard dose among MINOCA patients [128]. Because of the diverse etiology and prognosis, the optimum MINOCA candidates for anti-platelet treatment should be selected according to the underlying etiology. Thereby, when plaque rupture is suspected or diagnosed as a cause of MINOCA, DAPT is recommended for 1 year, and single antiplatelet therapy is recommended for lifelong therapy [129].

### 6.3. Etiological Therapy

In the case of thromboembolism, coronary thrombi or emboli can result from a variety of thromboembolic disorders, including (1) acquired thromboembolic causes (left ventricular thrombus, prosthetic heart valves, atrial fibrillation, etc.) and (2) hereditary thromboembolic causes [protein C/S and antithrombin deficiency, factor V Leiden, etc.] [21,130,131]. Once diagnosed, treatment schemes should be individualized depending on the identified MINOCA cause according to the international guidelines [132].

The etiological therapy for CAS-induced MINOCA includes calcium-channel blockers (CCB) and nitrates for secondary prevention [133]. The combination of pharmaceutical therapy along with smoking cessation in smokers yields symptom relief in a significant proportion of patients with frequent episodes of CAS. Montone et al. [31] found that MINOCA patients who had positive screening for CAS stimulation and received CCB had a better prognosis than their control counterparts.

In the case of SCAD, this is usually accompanied by intraluminal complications. If there is no obvious blood flow obstruction, conservative treatment is generally recommended because coronary intervention and stenting may exaggerate dissection and expand the original range of the lesion. For this purpose, pharmaceutical therapy is preferred over percutaneous revascularization, unless there is hemodynamic instability. The cornerstone of pharmaceutical therapy is aspirin and β-blockers [134]. The latter has been associated with a lower risk of recurrent SCAD in a large cohort study (HR, 0.39; 95% CI, 0.19–0.78; *p* = 0.008) [135]. For those undergoing percutaneous coronary intervention and stenting, DAPT is highly recommended [136]. Lifelong therapy with a low dose of aspirin is also recommended. Since its inconspicuous appearance is easily missed, and is not associated with atherosclerotic disorders, some researchers have not suggested traditional statin therapy [39].

In the case of CMVD, first-line anti-anginal therapy with β-blockers, CCBs and nitrates should be used, given their vasodilatory effect [137]. It is possible that the non-dihydropyridine members (diltiazem, verapamil) with a heart-rate-lowering effect may be a more favorable choice for angina relief. Ivabradine may play a role as a second-line agent, particularly in patients unable to tolerate β-blockers/non-dihydropyridine CCBs or those who do not achieve an adequately controlled heart rate on maximally tolerated treatment because of its ability to lower the heart rate [138].

**Figure 2:**
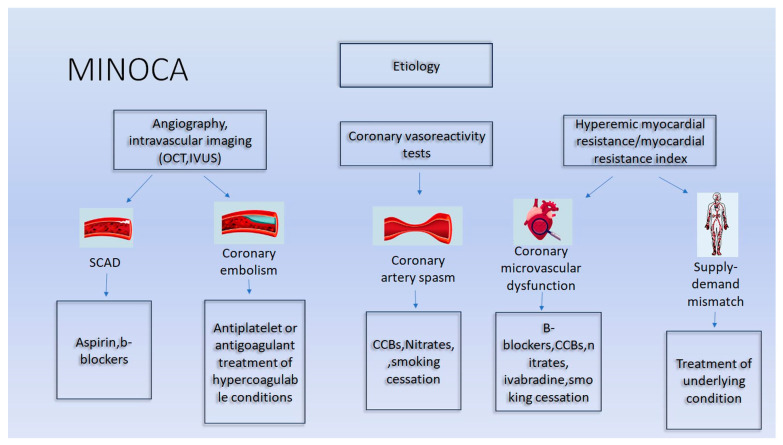
Therapeutic algorithm of MINOCA based on the underlying causes (atherosclerotic and non-atherosclerotic). CCB, calcium channel blockers; IVUS, intravascular ultrasound; oct, optical coherence tomography.

Additional strategies may also play a role in chronic pain alleviation, e.g., antidepressants. Alterations in lifestyle including weight loss, smoking cessation, a high-fiber diet, increased consumption of fruits and vegetables, and sport are also beneficial for the prognosis of MINOCA patients [139]. Smoking cessation has been recognized as the cornerstone therapy of CMVD [140]. An indicative pharmaceutical algorithm of MINOCA based on underlying causes is presented in Figure 2.

## 7. The Added Value, the Clinical Applications and Future Perspectives of Pathophysiology-Based MINOCA Management

Regarding the increasing number of papers about MINOCA entities, the present narrative review outlines the following aspects, proposing a significant point of view:

- After the initial diagnosis or the raised suspicion of MINOCA, a pathophysiological approach should be tailored to suit the diagnostic investigation and the etiological therapy. Ιt is impossible to apply uniform management for all cases of MINOCA, since its pathophysiology is diverse.
- The current narrative review makes a critical appraisal of all available diagnostic modalities for MINOCA investigation and their prognostic value.
- This review provides an extensive description of the potential role of established and novel circulating biomarkers in the diagnosis and risk stratification of patients with MINOCA. Notably, genetic factors and metabolomics could be a significant novel tool for both diagnosis and prognosis.

From the clinical perspective, good knowledge of the diagnostic modalities regarding their strengths and weaknesses will aid cardiologists during the decision-making process. The emerging role of biomarkers and their interpretation in the MINOCA setting will guide therapy and has the potential to provide an estimation of prognosis. Most importantly, the association of diagnostic findings with prognosis has been used in other cardiovascular diseases to determine the intensity of therapy. A similar approach is proposed in the present narrative review, which will unambiguously increase the effectiveness of proposed therapy targeting specific underlying causes. However, the prognostic value of available pharmaceutical therapy is still under investigation by long-term prospective studies.

Unfortunately, there is a lack of data regarding tailored therapies for MINOCA. However, there is a growing number of ongoing studies. The multicenter randomized PROMISE trial aims to apply the principles of precision medicine in patients hospitalized with MINOCA [141]. In particular, the researchers will comparatively examine a standard diagnostic and therapeutic approach of ACS versus an individualized diagnostic approach targeting causative reasons of MINOCA and tailored therapy. Another multicenter randomized trial will assess the impact of escalating doses of eplerenone on natriuretic peptides along with a long list of other outcomes, like biomarkers of vascular inflammation, quality of life, health economics, etc. [142]. Moreover, therapeutic studies investigating pharmaceutical or interventional therapies in MINOCA patients will shed more light on the underlying mechanisms and their impact on prognosis.

## 8. Conclusions

MINOCA is an increasingly recognized clinical entity associated with an increased risk of MACE among patients presenting with AMI [143]. Prompt diagnosis including multimodality imaging and biomarkers is of utmost importance to uncover the underlying cause. Treatment, though often empirical, is paramount and can be effective for symptom amelioration once the cause is established. Several prognostic factors of morbidity and mortality in AMI-CAD patients have been identified to correlate with MINOCA patients, especially inflammatory markers. However, more investigation will shed light on the clinical course of MINOCA. A significant knowledge gap persists regarding the diagnostic methods and management of patients with MINOCA. Therefore, future research should focus on those two domains following a pathophysiological approach, as follows: (a) a multimodality diagnostic approach should be employed for prompt recognition of MINOCA; (b) targeted evidence-based treatment should be instituted in order to prevent MACE and improve survival. Overall, the multifaceted nature, the demanding diverse diagnostic tools and the not fully clarified therapeutic strategies make MINOCA management a significant clinical challenge.

## Figures and Tables

**Table 1:** Methods for MINOCA diagnosis and prognosis.

Methods	Parameters	Diagnosis	Prognosis
**Echocardiography**	- Myocardial function - WΜA - CFR	- Causes of myocardial injury - Differential diagnosis of other non-cardiac diseases - CMVD	- Initial assessment tool
**Invasive Coronary Angiography**	- Coronary artery stenosis - Coronary flow rates - Epicardial coronary vasospasm	- Coronary artery visualization - SCAD - Vasospasm and microvascular dysfunction - Coronary artery bridging	- Poorer outcomes in non-obstructive coronary atherosclerotic plaques - Worse prognosis in positive provocative testing for coronary spasm
**Intravascular Ultrasound**	- 360-degree imaging of coronary arteries	- Detailed characterization of coronary lesions	- Poorer prognosis in complex lesions
**Optical Coherence Tomography**	- Visualization of luminal and superficial lesions	- High-resolution imaging for precise lesion detection (plaque disruption, SCAD, and distal embolization)	- Poorer prognosis in disrupted lesions
**Fractional Flow Reserve and Instantaneous Wave-Free Ratio**	- Pressure measurements	- Functional significance of epicardial stenosis - Microvascular function with CFR and IMR	- Risk stratification in specific cases
**Coronary Computed Tomography Angiography**	- Plaque characteristics - pFAI	- Identification of extracardiac structures - Lesion changes - Differentiation coronary vs. non-coronary origin of MINOCA	- Elevated pFAI values indicate load of inflammation
**Cardiac Magnetic Resonance**	- Myocardial perfusion - Ventricular function - T2 and LGE sequences	- Differentiation ischemic vs. non-ischemic injuries - Identification myocardial edema, fibrosis, and microvascular obstruction	- Prognostic value of LGE, MPI and MPRI

CMVD, coronary microvascular dysfunction; CFR, Coronary Flow Reserve; IMR, index of microcirculatory resistance; LGE, late gadolinium enhancement; MINOCA, myocardial infarction with non-obstructive coronary arteries; MPI, Myocardial Perfusion Imaging; myocardial perfusion reserve index; pFAI, Perivascular Fat Attenuation Index; SCAD, Spontaneous Coronary Artery Dissection; WMA, wall motion abnormalities.

**Table 2:** Biomarkers associated with MINOCA diagnosis and prognosis.

Biomarkers	Mechanism	Changes in MINOCA	Prognostic value
**Troponin (cTnI and cTnT)**	Myocardiocytes destroy	↑ cTn levels > 99th percentile: myocardial injury	Lower peak values in MINOCA Limited prognostic utility
**C-reactive protein (CRP)**	Inflammation	↑ in MINOCA	↑ risk of all-cause mortality and MACE
**P-selectin glycoprotein ligand-1 (PSGL-1)**	Inflammation	Discriminates between MINOCA, AMI-CAD and healthy controls	Poorer prognosis in MINOCA patients
**Interleukin 6 (IL-6)**	Inflammation, CMVD, CAS	↑ in CMVD and CAS	↑ adverse cardiovascular outcomes
**Soluble urokinase-type plasminogen activator receptor (suPAR)**	Inflammation	↑ in MINOCA vs. healthy controls	↑ adverse cardiovascular outcomes
**N-terminal pro-B-type natriuretic peptide (NT-proBNP)**	Endo-cardiac pressure	↑ in MINOCA and AMI-CAD vs. healthy controls	Unknown
**Lipoprotein (a) (Lp(a))**	Lipid metabolism	↑ in MINOCA	Poorer prognosis in MINOCA patients
**Visfatin**	Inflammation, endothelial function	↑ in MINOCA and type 2 diabetes mellitus	Unknown
**Placental growth factor (PlGF)**	Endothelial function, inflammation, angiogenesis	↑ in MINOCA	Unknown
**Fractalkine (CX3CL-1/FKN)**	Plaque formation, inflammation, endothelial dysfunction	↑ in MINOCA	Unknown
**White blood cell count to mean platelet volume ratio (WMR)**	Inflammation	↑ in MINOCA	↑ risk of MACE
**Neutrophil-to-platelet ratio (NPR)**	Inflammation	↑ in MINOCA	↑ risk of MACE
**Platelet-to-lymphocyte ratio (PLR)**	Inflammation	↑ in MINOCA	↑ risk of MACE
**Neutrophil-to-lymphocyte ratio (NLR)**	Inflammation	↑ in MINOCA	↑ risk of MACE
**Uric acid**	-	↑ in MINOCA	↑ adverse outcomes and MACE
***HMOX1 gene***	Stress-induced protective enzymes act against myocardial ischemia	Long promoter guanine-thymine repeats	↑ adverse outcomes and heart dysfunction
**ET-1 (Endothelin-1)**	Regulation vascular tone and proliferation	↑ in MINOCA	Association with CMVD
**TGF-β (Transforming Growth Factor-β)**	endothelial cell dysfunction and vascular permeability	↑ in MINOCA	Association with SCAD in women

CMVD, coronary microvascular dysfunction; CAS, coronary artery spasm; HMOX1, Heme Oxygenase 1; MACEs, major adverse cardiovascular events; AMI-CAD, acute myocardial infarction with coronary artery disease; MINOCA, myocardial infarction with non-obstructive coronary arteries; SCAD, Spontaneous Coronary Artery Dissection.
